# Supplementary material for: Rapid High-Resolution Analysis of Polysaccharide-Lignin Interactions in Secondary Plant Cell Walls Using Proton-Detected Solid-State NMR
Source: Anal Chem. 2025 Aug 15;97(33):18046–54. doi: 10.1021/acs.analchem.5c02059 (PMC12392260; doi:10.1021/acs.analchem.5c02059)
Supplement: Supplementary file 1 [file ac5c02059_si_001.pdf]

Supplementary Information

Rapid High-Resolution Analysis of Polysaccharide-Lignin Interactions in Secondary Plant Cell Walls Using Proton-Detected Solid-State NMR

Peng Xiao[1‡], Jayasubba Reddy Yarava[1‡], Debkumar Debnath[1], Priya Sahu[1], Yifan Xu[1], Li Xie[1], Daniel Holmes[1], Tuo Wang[1]*

[1] Department of Chemistry, Michigan State University, East Lansing, MI 48824, USA

Table of Contents

Supplementary Text S2

  Abbreviations used in maintext and SI S2

  Detailed information of experimental setup S3

Figure S1. Validation of spectrum assignment using DEEP picker S4

Figure S2. Through-space lignin-carbohydrates interactions in eucalyptus S5

Table S1. NMR experimental parameters S6

Table S2. ^{1}H and ^{13}C chemical shifts for the rigid carbohydrates in eucalyptus S7

Table S3. ^{1}H and ^{13}C chemical shifts for the rigid lignins in ecucalyptus S8

Supplementary References S9

SUPPLEMENTARY TEXT

Abbreviations used in maintext and SI

Ac, Acetyl; CP, cross polarization; DQ, double-quantum; DSS, Sodium trimethylsilylpropanesulfonate; GalA, galacturonic acid; GlcA, glucuronic acid; MAS, magic-angle spinning; MISSISSIPI, multiple intense solvent suppression intended for sensitive spectroscopic investigation of protonated proteins; NMR, nuclear magnetic resonance; rf, radio frequency; OMe, methoxyl; RFDR, radio frequency-driven recoupling; slpTPPM, swept low power two-pulse phase modulation; SPINAL-64, small phase incremental alteration, with 64 steps; TMS, tetramethylsilane; TOCSY: total correlation spectroscopy; WALTZ-16: wideband alternating-phase low-power technique for zero-residual splitting; Xn^{2f}, two-fold xylan; Xn^{3f}, three-fold xylan; Xyl, xylose.

Detailed information of experimental setup

Uniformly ^{13}C-labeled mature stems of eucalyptus (*Eucalyptus grandis*) were grown in ^{13}C-enriched (97 atom%) closed-atmosphere chambers for 16 weeks at IsoLife (Wageningen, The Netherlands)[1]. The debarked plant stems were finely cut into small pieces using a razor, and packed into a 1.3 mm MAS rotor for solid-state NMR analysis. All solid-state NMR experiments were performed on a Bruker Avance-NEO 600 MHz (14.1 T) spectrometer equipped with a 1.3 mm triple-resonance HCN probe, operating at a MAS rate of 60 kHz. ^{13}C chemical shifts were externally referenced using the tetramethylsilane (TMS) scale, with adamantane methylene resonance set at 38.48 ppm, while ^{1}H chemical shifts were referenced to sodium trimethylsilylpropanesulfonate (DSS) at 0 ppm. The cooling gas temperature was maintained at 250 K, with an estimated sample temperature of approximately 296 K, accounting for heating effects from fast MAS.

Short-range (predominantly one-bond) ^{1}H-^{13}C correlations were acquired using a 2D hCH experiment with a short second CP contact time setting to 100 µs. Through-bond ^{13}C-^{13}C connectivity was established using a 3D hCCH-TOCSY (total correlation spectroscopy) experiment[2], employing a 15-ms WALTZ-16 (wideband alternating-phase low-power technique for zero-residual splitting) mixing[3] with a radiofrequency (rf) field strength of 21.4 kHz. Through-space ^{1}H-^{1}H interactions were probed in eucalyptus using 2D hChH and 3D hCHH experiments,

incorporating RFDR-XY16 (radio frequency-driven recoupling) mixing sequences[4, 5] with recoupling times ranging from 0.133 ms to 0.8 ms. Water suppression for all experiments was achieved using the MISSISSIPPI (Multiple Intense Solvent Suppression Intended for Sensitive Spectroscopic Investigation of Protonated Proteins) sequence[6], with a 100 ms duration and an rf field strength of 15.4 kHz.

Heteronuclear dipolar decoupling was applied using slpTPPM (swept low-power two-pulse phase modulation) decoupling[7] at 13.5 kHz rf power on the ^{1}H channel during t_1 evolution in hCH and 2D/3D hCHH RFDR sequences. WALTZ-16 decoupling at 10 kHz was applied on the ^{13}C channel during direct detection of ^{1}H chemical shift evolution in the 2D hCH, 2D hChH, and 3D hCHH experiments. No decoupling was applied during the indirection dimension of ^{1}H chemical shift evolution, instead a π pulse was implemented for refocusing. For 3D hCCH TOCSY, slpTPPM decoupling was applied during t_1 and t_2 evolution on the ^{1}H channel, while WALTZ-16 decoupling at 10 kHz rf power was applied on the ^{13}C channel during direct ^{1}H detection. The 90° pulse lengths were 2.5 μs for ^{1}H (100 kHz rf power) and 5 μs for ^{13}C (50 kHz rf power). ^{1}H-^{13}C cross-polarization (CP) was performed under the double-quantum (n = +1) Hartmann-Hahn condition, with rf powers of 49.7 kHz for ^{1}H and 10 kHz for ^{13}C, at an MAS rate of 60 kHz.

In pulse sequence notation, uppercase letters denote nuclei that undergo frequency evolution, whereas lowercase letters indicate nuclei for which frequency evolution is omitted. For example, in the 2D hChH sequence, the lowercase "h" at the beginning and in the middle signifies that ^{1}H frequency evolution is not measured, "C" denotes ^{13}C frequency evolution in the t_1 dimension (δ_1), and the final "H" corresponds to ^{1}H detection in t_2 (δ_2). Similarly, in the 3D hCHH sequence, the initial lowercase "h" indicates that ^{1}H frequency evolution is omitted, "C" denotes of ^{13}C frequency evolution in t_1 (δ_1) dimension, the first "H" represents ^{1}H frequency evolution in t_2 (δ_2) and the second 'H' corresponds to 1H detection in t_3 (δ_2). 2D and 3D datasets were acquired using the States-TPPI method[8]. Detailed experimental parameters are provided in **Tables S1,** while the assigned ^{13}C and ^{1}H chemical shifts of carbohydrate and lignin polymers are listed in **Tables S2 and S3**.

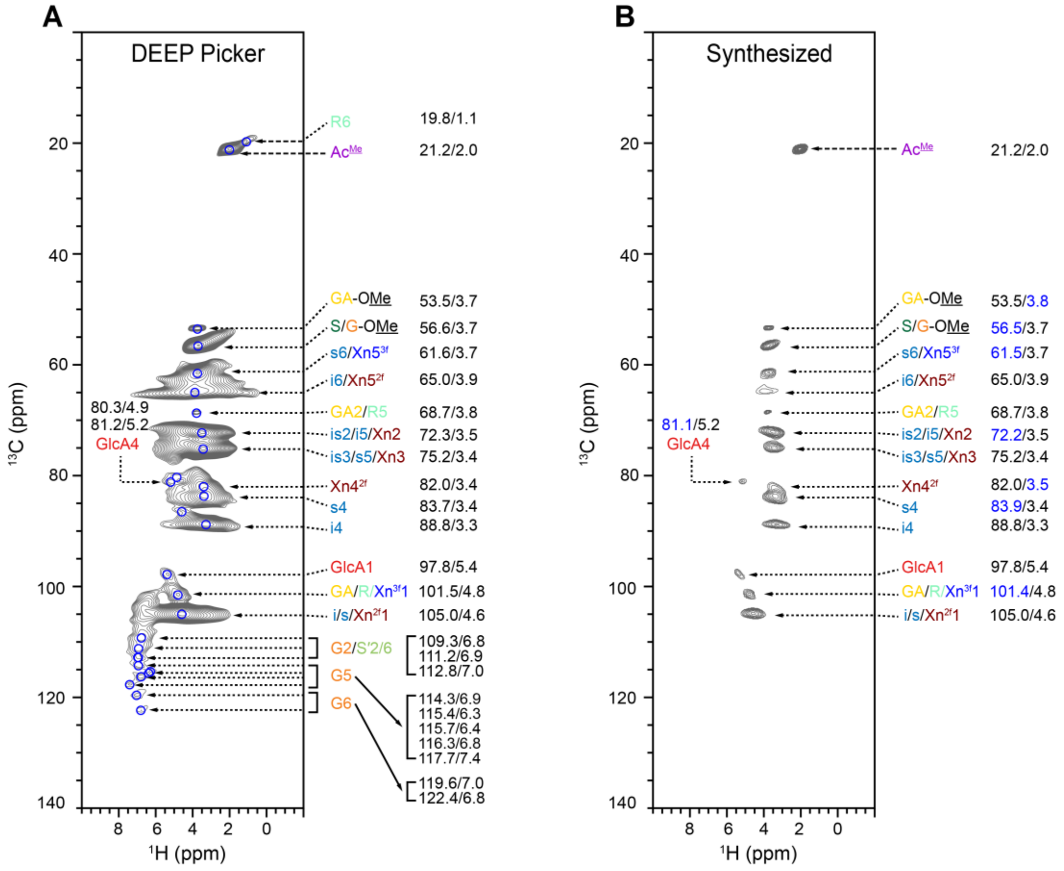

Figure S1. Validation of spectrum assignment using DEEP picker. The same 2D hCH spectrum with a short 0.1 ms ^{13}C-^{1}H CP contact time is plotted for spectrum assignment using (**A**) DEEP picker method and (**B**) Synthesized approach. In (**A**), the contour level cutoff is at 4.8 times root-mean-square of the noise. Blue circles mark the local maxima identified by DEEP picker classical mode, with their corresponding chemical shifts listed on the side. In (**B**), each peak is plotted separately with the contour level cutoff at its FWHM and reconstructed on the same spectrum. The identified chemical shifts that deviate from those in (**A**) are highlighted in blue, with deviations typically less than 0.2 ppm.

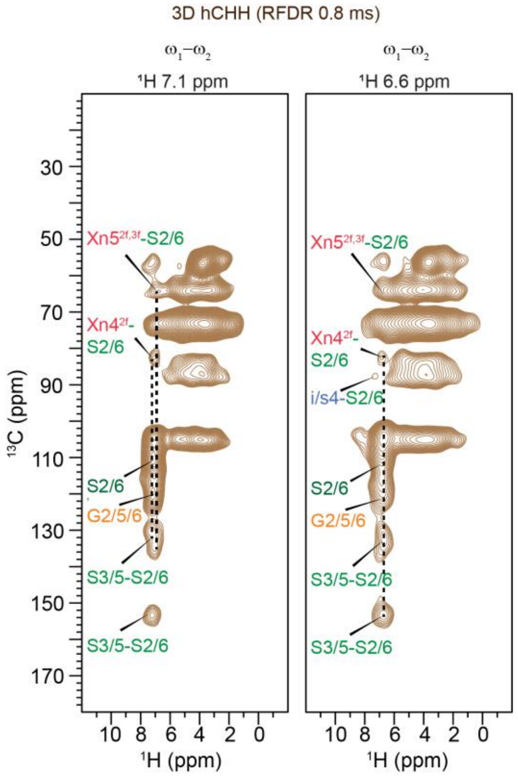

Figure S2. Through-space lignin-carbohydrates interactions in eucalyptus analyzed by ^{1}H-^{13}C (ω_1-ω_2) planes of 3D hCHH spectrum. 2D strips of ^{1}H-^{13}C (ω_1-ω_2) were extracted at different proton chemical shifts in (ω_3) from the 3D hCHH RFDR experiment with mixing time of 0.8 ms. The spectra were measured on a 14.1 T spectrometer with a MAS rate of 60 kHz.

Table S1. NMR experimental parameters. τ_{cp1} and τ_{cp2}: the contact times for the first (hC) and the second (Ch and CH) CP, respectively. NS: number of scans; D1 = recycle delay between scans. TD1/2/3 and AQ1/2/3: total data points and chemical shift evolution time for dimensions 1/2/3, respectively.

Expt.	CP (μs)		NS	D1 (s)	TD1	TD2	TD3	AQ1 (ms)	AQ2 (ms)	AQ3 (ms)	WALTZ (ms)	RFDR (μs)	Expt. Time	Sample
	τ_{cp1}	τ_{cp2}												
2D hCH	1200	100	16	2	384 (^{13}C)	2000 (^{1}H)	-	6.4	10.0	-	-	-	3h39m	Eucalyptus
	2000	50	16	2	384 (^{13}C)	2000 (^{1}H)	-	6.4	12.2	-	-	-	3h41m	
2D hChH	2000	500	32	2	1600 (^{1}H)	256 (^{13}C)	-	4.3	13.6	-	-	133.3<br>266.7<br>800.0	4h52m	
3D hCHH	2000	500	16	2	84 (^{13}C)	84 (^{1}H)	1600 (^{1}H)	1.4	1.4	13.6	-	800.0	2d18h35m	
3D hCCH TOCSY	2000	100	8	2	128 (^{13}C)	128 (^{13}C)	1764 (^{1}H)	2.1	2.1	14.9	15	-	3d5h51m	

Table S2. ^{1}H and ^{13}C chemical shifts for the rigid carbohydrates in Eucalyptus. ^{1}H is on DSS scale and ^{13}C is on TMS scale. Unidentified sites are indicated as "-". Not applicable ones are indicated with "/". Ambiguous assignments (i.e. no previous data to confidently assign to a certain molecule or site) are underlined. The abnormal proton shifts in xlyan are due to potential acetylation and are indicated with "*".

	C1/H1	*C2/H2*	*C3/H3*	*C4/H4*	*C5/H5*	*C6/H6*	*OMe*	*AcMe*
	105.5/4.58	72.2/3.51	75.2/3.42	88.5/3.28	72.2/3.51	65.0/3.90	/	/
i	105.5	71.7	-	89.0/3.53	71.7	65.3	/	/
	104.6	-	-	87.2/3.1	-	65.5/3.50	/	/
	103.2/4.95	-	-	86.6	-	-	/	/
i/s	-	-	-	86.0/4.38	-	65.3	/	/
	-	-	-	86.9/4.19	-	68.0	/	/
	105.4/4.58	72.2/3.51	75.2/3.42	84.8/3.80	75.2/3.42	61.5/3.72	/	/
s	104.2/5.12	71.1/3.39	75.5	84.7/2.56	75.5	62.0/3.34	/	/
	-	72.0	74.8	85.2/3.72	74.8	64.8/3.70	/	/
	105.0/4.58	72.2/3.51	75.2/3.42	81.8/3.48	63.5/3.90	/	/	21.2/2.02
Xn2f	105.0	73.5	74.6	82.7/3.72	63.6	/	/	-
	103.8/5.16*	72.8/4.02*	74.4/4.88*	82.2/4.62*	62.4/4.02*	/	/	-
	-	73.4/3.90	73.4/3.90	80.5/3.27	64.9/3.62	/	/	21.2/2.02
Xn	-	73.2	73.2	80.3/3.92*	64.2/3.65	/	/	-
	-	-	-	80.2/5.1*	61.1	/	/	-
	102.2/4.58	73.3/5.08*	74.4/4.09*	78.8/3.63	63.9/3.30	/	/	21.2/2.02
	-	71.34/3.70		78.3	64.3/3.32	/	/	-
Xn3f	-	-	74.2/4.89*	78.9/3.65	65.0	/	/	-
	-	-	-	79.2/3.63	64.6	/	/	-
	102.3	-	-	77.5/4.73*	-	/	/	-
	101.9/5.12	71.1	73.5	77.8	-	/	/	-
	98.5/5.30	72.7	75.7	81.5/3.46	67.9/4.72	176.8	59.6/3.58	/
	97.8/4.8	72.9	75.2	81.6/4.25	66.4/4.4	177.6	-	/
Me-GlcA	98.2	-	-	81.8/2.67	69.5/3.84	-	-	/
	98.1/4.81	-	-	83.8	70.5	-	-	/
	98.1/4.73	-	-	80.3	70.7	-	-	/
	100.8/5.14	68.0/3.63	-	78.5/4.35	70.5/3.46	171.2	53.5/3.75	21.2/2.02
	101.4/4.81	68.8/3.79	-	79.0/4.34	71.3/3.27	170.5	-	-
GalA	100.8	69.4/4.18	-	79.8/4.68	-	169.6	-	-
	100.8/3.99	-	-	75.7	-	-	-	-
	99.6/4.7	69.9	-	-	-	-	-	-
Rha	-	-	-	-	-	17.6/1.15	/	/
	-	-	-	-	-	19.4/0.91	/	/

Note: The OMe, CH_3 (AcMe and R6) and CO (GalA6 and Me-GlcA6) chemical shifts were identified in 2D hCH and 2D hChH RFDR spectra.

Table S3. ^1H and ^{13}C chemical shifts for the rigid lignin in Eucalyptus. ^1H is on DSS scale and ^{13}C is on TMS scale. Remote protons are indicated with "*".

Lignin site	*^{13}C*	*^1H*
OMe	56.5	3.70
S2/6	101.5-106.5	6.00-7.26
G2<br>*S'2/6*	109.2	6.79
	111.2	6.98
	112.8	6.94
G5	114.3	6.90
	115.2	6.23/7.31
	116.3	6.79
	117.7	7.44
G6	119.6	7.06
	120.3	6.03
	122.1	6.72
S1/G1	131.1	6.20*
	132.1	6.94*
	134.1	6.72*
G3/4	147.7	7.96*
S3/5	152.2	7.09*
	152.6	6.49*
	153.1	7.65*
	153.8	6.76*

Supplementary References

(1) Kirui, A.; Zhao, W.; Deligey, F.; Yang, H.; Kang, X.; Mentink-Vigier, F.; Wang, T. Carbohydrate-aromatic interface and molecular architecture of lignocellulose. *Nat. Commun.* **2022**, *13*, 538.

(2) Andreas, L. B.; Jaudzems, K.; Stanek, J.; Lalli, D.; Bertarello, A.; Le Marchand, T.; Cala-De Paepe, D.; Kotelovica, S.; Akopjana, I.; Knott, B.; et al. Structure of fully protonated proteins by proton-detected magic-angle spinning NMR. *Proc. Natl. Acad. Sci. USA* **2016**, *113*, 9187-9192.

(3) Shaka, A.; Keeler, J.; Frenkiel, T.; Freeman, R. An improved sequence for broadband decoupling: WALTZ-16. *J. Magn. Reson.* **1983**, *52*, 335-338.

(4) Nishiyama, Y.; Zhang, R.; Ramamoorthy, A. Finite-pulse radio frequency driven recoupling with phase cycling for 2D 1H/1H correlation at ultrafast MAS frequencies. *J. Magn. Reson.* **2014**, *243*, 25-32.

(5) Bennett, A. E.; Rienstra, C. M.; Griffiths, J. M.; Zhen, W.; Lansbury, P. T.; Griffin, R. G. Homonuclear radio frequency-driven recoupling in rotating solids *J. Chem. Phys.* **1998**, *108*, 9463-9479.

(6) Zhou, D. H.; Rienstra, C. M. High-performance solvent suppression for proton detected solid-state NMR. *J. Magn. Reson.* **2008**, *192*, 167-172.

(7) Lewandowski, J. R.; Sein, J.; Sass, H. J.; Grzesiek, S.; Blackledge, M.; Emsley, L. Measurement of Site-Specific 13C Spin−Lattice Relaxation in a Crystalline Protein. *J. Am. Chem. Soc.* **2010**, *132*, 8252-8254.

(8) Marion, D.; Ikura, M.; Tschudin, R.; Bax, A. Rapid recording of 2D NMR spectra without phase cycling. Application to the study of hydrogen exchange in proteins. *J. Magn. Reson.* **1989**, *85*, 393-399.

(9) Lesage, A.; Auger, C.; Caldarelli, S.; Emsley, L. Determination of through-bond carbon-carbon connectivities in solid-state NMR using the INADEQUATE experiment. *J. Am. Chem. Soc.* **1997**, *119* (33), 7867-7868. DOI: Doi 10.1021/Ja971089k.

(10) Lesage, A.; Bardet, M.; Emsley, L. Through-Bond Carbon−Carbon Connectivities in Disordered Solids by NMR. *J. Am. Chem. Soc* **1999**, *121* (47), 10987-10993.
